# Impact of Winemaking Techniques on the Phenolic Composition and Antioxidant Properties of Touriga Nacional Wines

**DOI:** 10.3390/molecules30071601

**Published:** 2025-04-03

**Authors:** Zélia Branco, Filipa Baptista, Jessica Paié-Ribeiro, Irene Gouvinhas, Ana Novo Barros

**Affiliations:** 1 Centre for the Research and Technology of Agro-Environmental and Biological Sciences (CITAB), Institute for Innovation, Capacity Building and Sustainability of Agri-Food Production (Inov4Agro), University of Trás-os-Montes and Alto Douro, Quinta de Prados, 5000-801 Vila Real, Portugal; mzbranco@sapo.pt (Z.B.); fbaptista@utad.pt (F.B.); igouvinhas@utad.pt (I.G.); 2 Veterinary and Animal Research Centre (CECAV), University of Trás-os-Montes e Alto Douro, 5000-801 Vila Real, Portugal; jessicapaie@utad.pt

**Keywords:** wine, Touriga Nacional, phenolic composition, antioxidant capacity, RP-HPLC-DAD-ESI-MS/MS

## Abstract

The Touriga Nacional grape variety is renowned in Portuguese red wines for its intense color and aromatic complexity, largely attributed to its rich phenolic composition. Several factors influence the phenolic profile of wines, including edapho-climatic conditions, grape variety, and winemaking techniques such as fermentation, maceration, barrel aging, and maturation. In this study, the technique for winemaking was the only controlled variable, allowing for a specific evaluation of its impact on phenolic composition and antioxidant capacity. Ten single-varietal Touriga Nacional wine samples from the 2019 vintage, produced in the Cima Corgo sub-region of the Douro by different wineries, were analyzed. The phenolic composition was determined using colorimetric methods to quantify total phenols, *ortho*-diphenols, flavonoids, anthocyanins, and tannins. Antioxidant capacity was assessed through the 2,2-diphenyl-1-picrylhydrazyl (DPPH), 2,2′-Azino-bis (3-ethylbenzothiazoline-6-sulfonic acid) diammonium salt (ABTS), and Ferric Reducing Antioxidant Power (FRAP) assays. Since all wines shared the same grape variety, region, and harvest year, the fermentation technique was the main differentiating factor, enabling a direct comparison of its influence on phenolic extraction and antioxidant properties. Additionally, Reversed-Phase High-Performance Liquid Chromatography with Photodiode Array Detection coupled with Mass Spectrometry (RP-HPLC-DAD-ESI-MS/MS) was employed to identify and quantify individual phenolic compounds. This study highlights the key role of winemaking techniques in modulating the phenolic composition and antioxidant potential of Touriga Nacional wines.

## 1. Introduction

Wine is an alcoholic beverage derived from the fermentation of grape must. In the European Union, its production is strictly regulated, requiring the partial or complete alcoholic fermentation of fresh grapes or grape must [1]. The wine industry holds substantial economic and cultural significance worldwide [2,3]. Portugal ranks as the tenth-largest wine producer globally [4] and, as part of the Old-World winemaking tradition, benefits from centuries of viticultural expertise that reinforce its economic relevance [5,6]. Among its indigenous grape varieties, Touriga Nacional stands out as a key cultivar in the Douro Demarcated Region (DDR), the world’s oldest regulated wine appellation [7]. From a viticultural perspective, Touriga Nacional exhibits a prostrate or semi-erect growth habit, posing challenges in vineyard management, particularly in vigorous growth conditions. Its high vegetative vigor makes it susceptible to wind damage and diseases such as scoriosis. To optimize its development, grafting onto highly vigorous rootstocks and planting in excessively fertile or humid soils should be avoided, as these conditions may exacerbate shoot breakage and delay phenolic ripening—an essential factor for producing high-quality wines [7,8,9,10,11,12,13]. From an oenological standpoint, Touriga Nacional is highly versatile, capable of yielding both premium table wines and fortified wines, such as Port, provided that it is harvested at the onset of overripeness [4,10].

**Figure 1:**
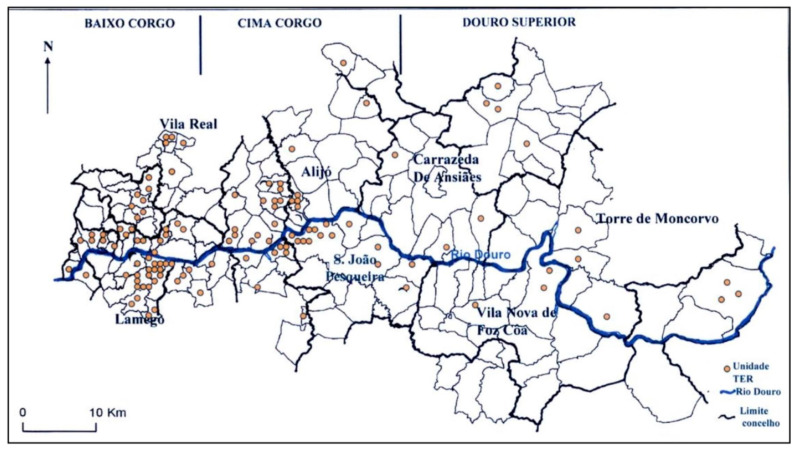
Map of the limitations of the Douro Demarcated Region—RDD [16].

The DDR, located in the Douro River basin, extends from Barqueiros (Mesão Frio) to Mazouco (Freixo de Espada à Cinta), covering approximately 250,000 hectares, of which 24,600 hectares are dedicated to vineyards [14,15]. The region is divided into three sub-regions—Baixo Corgo, Cima Corgo, and Douro Superior—each distinguished by unique climatic and geographical characteristics, as illustrated in Figure 1.

This study characterizes 10 single-varietal Touriga Nacional wines from the Cima Corgo sub-region, vintage 2019, from different producers, highlighting phenolic composition and antioxidant capacity variations due to winemaking differences. Total phenols, *ortho*-diphenols, flavonoids, anthocyanins, and tannins were analyzed using colorimetric techniques. Antioxidant capacity was assessed via ABTS, DPPH, and FRAP methods. RP-HPLC-DAD-ESI-MS/MS was used for detailed phenolic profiling and regional marker identification. Finally, statistical methods correlated phenolic profiles with colorimetric data.

### The Role of Polyphenols in Wine: Extraction and the Impact of Winemaking Techniques

The extraction of polyphenols during winemaking techniques, such as crushing, can be unfavorable since several reactions may occur that involve polyphenols, mainly anthocyanins, catechins, and procyanidins, forming new polymeric pigments responsible for changes in the color of wines due to oxidation and enzymatic reactions. On the other hand, the extraction of polyphenols during winemaking can contribute to the greater stability of wines [17].

Moderate red wine consumption has been associated with reduced mortality from cardiovascular diseases [3,18].

Polyphenols, naturally present in grapes—the primary raw material for wine production—play a central role in this association. These compounds are secondary metabolites synthesized by the vine in response to biotic and abiotic stresses [19,20,21].

Phenolic compounds can be divided into two broad classes: non-flavonoids and flavonoids [22]. Non-flavonoids include phenolic acids (which are further divided into benzoic acids and cinnamic acids) and stilbenes [23]. Flavonoids are low molecular weight secondary metabolites, often linked to saccharides in the form of glycosides or aglycones, and are classified into six subclasses: flavonols, flavones, flavan-3-ols, flavanones, anthocyanins, chalcones, and isoflavones [24,25].

The extraction of polyphenols during winemaking techniques, such as crushing, can be challenging. Various reactions, especially involving anthocyanins, catechins, and procyanidins, may occur, leading to the formation of new polymeric pigments that change the wine’s color due to oxidation and enzymatic reactions. However, polyphenol extraction can also enhance wine stability [17].

**Figure 2:**
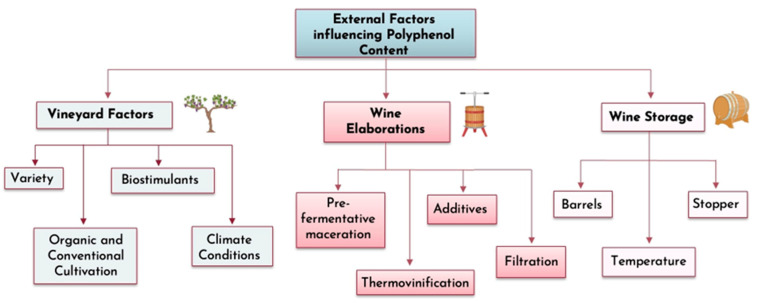
Summary diagram about the external factors selected to be studied and their influence on phenolic compounds in grape, must, and wine.

The quality and organoleptic properties of wine are significantly influenced by its phenolic content. The concentration of phenolic compounds is affected by a variety of internal and external factors, such as those in the vineyard, the winemaking process, and storage [26], as illustrated in Figure 2. Factors like temperature, additives, maceration duration, and filtration all influence phenolic retention [27,28].

Optimal grape ripeness leads to the accumulation of phenolic compounds and favorable qualitative changes in wine, such as reduced tannin astringency and increased volume and smoothness [29,30,31]. Aging processes further alter the phenolic composition, enhancing color and mouthfeel through polymerization. Barrel aging specifically affects polyphenol levels, with toasting reducing astringency, while cork permeability influences oxidation [32,33,34,35].

## 2. Results and Discussion

### 2.1. Phenolic Content

The quality and organoleptic properties of wine are impacted by its phenolic content. External factors such as vineyard conditions, winemaking processes, and storage significantly influence the concentration of phenolic compounds [26].

Regarding the quantification of total phenolic content (TPC), *ortho*-diphenols (ODC), and flavonoids (FC), results can be observed in Table 1.

Samples five and six exhibited significantly higher concentrations of *ortho*-diphenols (ODC) and flavonoids (FC) than the other samples. Specifically, sample five contained 3677.78 ± 166.70 mg GAE/L of ODC and 2115.22 ± 107.60 mg CE/L of flavonoids, while sample six had 3505.74 ± 168.23 mg GAE/L of ODC and 2154.11 ± 116.58 mg CE/L of flavonoids. In contrast, samples nine and ten presented the lowest values, with sample nine containing 518.28 ± 38.02 mg GAE/L of ODC and 730.44 ± 7.69 mg CE/L of flavonoids, and sample ten showing 431.66 ± 27.38 mg GAE/L of ODC and 707.73 ± 41.64 mg CE/L of flavonoids (Table 1).

Despite these elevated levels of specific phenolic compounds, the total phenolic content (TPC) values for samples five and six, measured using the Folin–Ciocalteu method, were unexpectedly lower—1029.28 ± 3.19 mg GAE/L and 1043.54 ± 78.68 mg GAE/L, respectively. These values were significantly lower than those observed in most other samples, except for sample one. Conversely, sample nine displayed the highest TPC, with a value markedly different from all the other samples (Table 1).

This apparent discrepancy suggests that the total phenolic content measured by the Folin–Ciocalteu method does not directly reflect the concentration of specific phenolic subgroups, such as *ortho*-diphenols and flavonoids [36,37]. The Folin–Ciocalteu reagent reacts with a wide range of reducing substances, and its response can be influenced by the presence of interfering compounds or by variations in the reactivity of different phenolics. Thus, the lower TPC values in samples five and six, despite their high ODC and FC levels, could indicate a lower presence of other phenolic classes, such as hydroxycinnamic acids, tannins, or stilbenes, which might contribute more significantly to the overall TPC in other samples, like sample nine.

Aging in wooden barrels is a key factor influencing the phenolic composition of wine. The extended aging of samples five and six may have contributed to the transformation of certain phenolics through oxidation, polymerization, and condensation reactions, altering their detectability in the Folin–Ciocalteu assay [38]. These processes impact anthocyanins, which convert into more stable oligomeric and polymeric pigments, modifying both the color and astringency of the wine. Additionally, flavonols undergo condensation reactions that affect their extractability. While aging can enhance wine complexity, it may also promote oxidation reactions, leading to a potential decrease in the concentration of simple phenolics. Factors such as temperature, barrel type, and oxygen exposure during aging further influence these changes [32,33,34,35,39].

Winemaking techniques also play a crucial role in shaping the phenolic profile. For instance, in sample five, the grapes were only lightly pressed after malolactic fermentation, which could have limited the extraction of certain phenolic compounds. Sample six, on the other hand, was fermented in a mill using traditional foot-treading, which enhances the contact between the liquid and the skins, typically increasing phenolic extraction [25]. Techniques such as traditional treading and pump-over (remontage) further influence phenolic retention, facilitating extraction while also preventing acetic bacteria development [40].

Sample eight, which exhibited consistently high phenolic values across all methods, may have benefited from maceration at higher temperatures. Temperature influences the kinetics of chemical reactions during aging, and the type of wood used in barrels can also affect polyphenol content [40]. Some studies suggest that the reuse of barrels may reduce polyphenol levels, but it remains unclear whether the high values observed in sample eight were solely due to maceration conditions or additional winemaking practices. Further clarification is needed regarding the influence of these factors.

Throughout vinification, phenolic content is shaped by multiple stages, including pre-fermentative maceration, thermovinification, the addition of chemical or natural additives, post-fermentative maceration, and filtration [26]. Methodological variations and the potential presence of interfering substances could explain the differences observed between samples.

Despite these variations, the phenolic content values reported in this study align with those found in the literature. For example, Vilela et al. [41] reported a TPC of 2290.00 ± 57.00 mg GAE/L and flavonoid content of 1858.00 ± 39.00 mg CE/L, while Paixão et al. [42] and Minussi et al. [43] found a TPC of 1724.00 ± 7.90 mg GAE/L. These comparisons reinforce the idea that phenolic composition is highly dependent on winemaking and aging conditions, as well as the methodology used for quantification.

### 2.2. Anthocyanins and Tannins Content

The quantification of total anthocyanins and tannins is presented in Table 2.

Regarding the quantification of total anthocyanins, sample five exhibited the highest value (556.00 ± 1.70 mg Malv/L), significantly different from the other samples. Samples two and three showed the lowest values (91.40 ± 8.77 and 94.40 ± 9.05 mg Malv/L, respectively), with no significant difference between them, but both were significantly lower than the other samples. The higher values in samples five and six can be attributed to their prolonged contact with wood [44,45]. The analysis of anthocyanins was performed in duplicate, not only due to limitations in sample availability but also considering the stability of these compounds and logistical challenges during the experimental process. While triplicate analyses would have been preferable to enhance data robustness, practical constraints prevented their execution. Nevertheless, the results obtained were consistent and provided a reliable assessment of the anthocyanin profile in the analyzed samples.

For tannins, sample three had the highest value (157.79 ± 6.62 mg EPI-CAT/L), significantly differing from the other samples. The lowest values were observed in samples four, five, six, and seven (67.05 ± 2.52, 67.90 ± 4.81, 77.23 ± 5.46, and 65.84 ± 3.29 mg EPI-CAT/L, respectively), with no significant difference between them. The high anthocyanin content in sample five can be attributed to its aging process. Aging and storage are key factors influencing the phenolic composition of wine, as these processes are affected by temperature, chemical reactions, wood type, and stoppers. During aging, phenolic compounds undergo several chemical reactions, such as the transformation of anthocyanins into more stable oligomeric and polymeric pigments, which impact both the color and astringency properties of the wine [46,47,48,49,50].

The high tannin content in sample three is attributed to its 32-day maceration process, as tannins are primarily found in the skins and seeds of grapes. This maceration process helps degrade the cell wall through the enzymatic action of pectinase, though it is influenced by temperature. For this reason, maceration is ideally conducted at low temperatures to prevent yeast growth and delay the onset of fermentation, as some phenolic compounds are more easily extracted in the absence of ethanol [51]. Another factor contributing to the elevated tannin content in sample three is that it was the only sample exposed to new wood, which contains higher tannin levels than aged wood. New wood has significantly more tannin, leading to greater extraction during the aging process and resulting in wines with higher tannin content. In fact, the choice of wood barrels for aging is influenced by both empirical knowledge and economic considerations. The roasting process plays a crucial role in shaping the final composition of the wood, as the application of heat causes chemical transformations and degradation of compounds. Typically, roasting leads to a reduction in astringency and an increase in aromatic substances [52,53,54,55,56].

The more or less prolonged contact of the must with solid grape parts, such as the skins, pulp, and seeds, after crushing and/or destemming, is called maceration. In traditional red winemaking, maceration takes place during fermentation and is intensified by the increase in temperature and alcohol content of the medium. Maceration partially extracts various phenolic compounds responsible for the color, aroma, and taste of red wines. It is, therefore, important to understand the factors that condition it: the state of ripeness, maceration time, temperature, mechanical actions, and alcohol content [40,57].

During the initial phase of maceration, in a medium-low ethanol concentration, anthocyanins are the first phenolic compounds to be extracted. As a result, the color intensity rises rapidly, usually reaching a maximum within a week. After this, the color intensity decreases due to adsorption by solid parts, yeasts, and the destruction of the colored combinations of anthocyanins with tannins. Unlike color intensity, the hue of wines increases with maceration time, evolving toward a relative decrease in red pigmentation and an increase in yellow pigmentation. Thus, the tannin content initially increases as the wine becomes richer in ethanol [40,58,59].

Compared to the literature, the values for anthocyanins reported by Vilela, A. [41] (804.00 ± 3.00 mg Malv/L) were higher than those observed in this study. This difference may be attributed to variations in winemaking methods, as significant discrepancies in the results were noted across the samples in this study. Anthocyanins constitute a major portion of flavonoids in red grapes, both in terms of quality and quantity. These compounds are primarily responsible for the color of grapes and wines, while 3-flavanols contribute to taste sensations, particularly astringency, and play an important role in the aging process of wine [40,41,42].

### 2.3. Impact of Maceration Temperature on Phenolic Compound Extraction and Wine Quality

The maceration temperature plays a crucial role in the extraction of phenolic compounds, particularly tannins, which are key contributors to the wine’s sensory properties. In this study, the variations in maceration temperature between samples five, three, and eight offer valuable insights into how temperature influences both the quantity and diversity of phenolic compounds in the final wine.

Sample 5, which underwent maceration at a lower temperature, likely experienced a slower and more controlled extraction of phenolic compounds, particularly tannins. This process tends to result in wines with softer, more refined tannins and a smoother mouthfeel, which are often desirable in high-quality wines. Lower maceration temperatures allow for greater preservation of delicate aromas while preventing excessive tannin extraction, leading to a more balanced wine profile [60].

In contrast, sample three, which was subjected to higher maceration temperatures, likely underwent a more intense extraction of tannins. High temperatures promote the breakdown of grape skins and seeds, releasing larger amounts of tannins into the wine. As a result, this may lead to a more astringent wine with an increased body, which could be perceived as either positive or negative, depending on the desired wine style. Higher maceration temperatures can also accelerate the release of anthocyanins, contributing to a more intense color but potentially resulting in a wine that is perceived as too tannic or aggressive if not properly balanced [61].

Sample eight, which was fermented at a higher temperature, could have benefited from enhanced extraction of both tannins and anthocyanins. The increased temperature likely facilitated the release of a wider variety of phenolic compounds, thereby enriching the wine’s aromatic profile and boosting its antioxidant capacity. However, if the maceration temperature exceeds optimal levels, it could lead to over-extraction, resulting in wines with harsh tannins and undesirable bitterness. Therefore, it is essential to carefully manage maceration temperatures to optimize the extraction of phenolic compounds without compromising the balance and overall sensory quality of the wine [60,62,63].

In conclusion, the temperature at which maceration is carried out is a critical factor in determining the phenolic composition of the wine. By adjusting maceration temperatures, winemakers can influence the extraction of tannins, anthocyanins, and other phenolic compounds, ultimately shaping the wine’s flavor, texture, and color. Understanding the relationship between temperature and phenolic extraction is essential for tailoring winemaking techniques to achieve the desired sensory characteristics and wine quality [64,65].

### 2.4. Chromatographic Analysis of Phenolic Compounds

In order to classify, authenticate, and trace wine samples, Reverse Phase High-Performance Liquid Chromatography Equipped with Photodiode Array with Electrospray Ionization Detection-Mass (RP–HPLC–DAD–ESI-MS/MS) was used to create the profile and fingerprint of each sample, allowing for more precise phenolic profiling. This makes it possible to characterize aspects, such as the wine’s region of origin. For this reason, coupling with Mass Spectrometry (MS) enables the creation of a database of discriminating characteristics [65,66,67].

**Figure 3:**
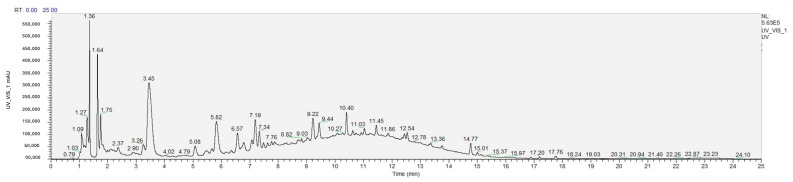
Representative chromatogram.

The phenolic compounds in wine samples were characterized using RP–HPLC–DAD–ESI-MS/MS. Table 3 provides information on the retention time and concentrations of phenolic compounds quantified. A total of 17 phenolic compounds were identified and quantified, covering classes such as stilbenes, hydroxynamic acids, flavan-3-ols, and flavonols. Figure 3 presents a representative chromatogram of the samples under study.

#### 2.4.1. Stilbenes

With regard to stilbenes, the compounds viniferin diglycoside and resveratrol tetramer were identified and quantified. This class of compounds was present in samples two, three, four, five, and seven, with sample four being the only one where resveratrol tetramer was detected. Sample five exhibited the highest concentration (75.83 ± 1.56 mg/L), which may be attributed to its extended aging period in wooden barrels. The literature suggests that phenolic compounds, such as resveratrol, are more stable and abundant in wines aged in wood. This stability is due to controlled exposure to oxygen and the presence of oak tannins, which can facilitate the formation of stable complexes [68].

The presence of resveratrol in smaller quantities contributes to the overall composition of red wines, with studies indicating that this compound is more abundant in wines that undergo vinification and prolonged aging [69].

#### 2.4.2. Hydroxycinnamic Acids

Regarding hydroxycinnamic acids, three phenolic compounds were identified and quantified: caftaric acid, caftaric acid-glucuronide, and chlorogenic acid, with caftaric acid being the most frequently detected of the three. This class was absent in samples seven and ten, while sample six showed the highest concentration (4.73 ± 0.09 mg/L). These acids are known to play a role in the color stability and antioxidant protection of wine. The variations observed between the samples can be attributed to differences in winemaking techniques, such as prolonged contact with grape skins or the extraction of these compounds during barrel aging. Chlorogenic acid was also detected in small amounts in samples one and eight. This particular compound is linked to antioxidant properties and contributes to the bitterness of the wine, influencing the sensory profile of younger wines [19].

#### 2.4.3. Flavan-3-Ols

Flavan-3-ols represented the class with the most phenolic compounds identified and quantified. These included procyanidin dimer digallate A-type isomer, proanthocyanidin dimer, proanthocyanidin tetramer, proanthocyanidin trimer, proanthocyanidin trimeric monogallate, (Epi)catechin-(epi)catechin gallate, (Epi)catechin-3-*O*-dihexoside, (Epi)catechin-(epi)gallocatechin gallate (EGCG), and (Epi)gallocatechin-(epi)catechin. These compounds were absent in samples two and nine, with the highest concentration found in sample six (30.14 ± 0.06 mg/L). Proanthocyanidins are known to contribute to the astringency of wine and are more prevalent in wines produced using winemaking methods that maximize contact with grape skins [70].

The presence of procyanidin dimers and other type B proanthocyanidins is associated with prolonged barrel aging, which facilitates the formation of complex compounds that enrich the wine’s tannin profile [71].

#### 2.4.4. Flavonols

In the flavonols class, three phenolic compounds were identified and quantified: myricetin-*O*-hexoside, esterified quercetin II, and esterified quercetin I. This class was present in only three of the ten samples—samples one, three, and seven—with the highest concentration found in sample three (3.39 ± 0.03 mg/L). Flavonols are important for color stability and have antioxidant properties. These compounds are often associated with wines that undergo skin contact, which enhances their extraction. Aging and storage are key factors influencing the phenolic content of wine, as these processes are affected by temperature, chemical reactions, wood, and stoppers. During aging, phenolic compounds undergo chemical reactions that contribute to the refinement of wine. In particular, anthocyanins transform into more stable oligomeric and polymeric pigments, which significantly impact color and astringency. Flavonols also participate in these reactions, particularly through condensation. Consequently, aging is essential to achieving the desired quality of the final product. However, this process can also lead to oxidation reactions, which may have undesirable effects. In general, younger wines contain higher levels of anthocyanins and simpler phenolic compounds, while older wines tend to have more stable polymeric pigments and a deeper color. The chemical changes during this phase are influenced by factors such as the initial concentration of phenolic and volatile compounds, pH, oxygen levels, acidity, and external factors like temperature, barrels, humidity, and light [33,34,35].

Esterified quercetin was detected in very small concentrations in only a few samples, such as one and seven, which could reflect specific winemaking techniques or the ripeness of the grapes.

HPLC analysis shows notable variations among the samples regarding both the diversity and quantity of phenolic compounds. Sample six stands out due to the highest number of compounds identified, which may be attributed to a winemaking process that facilitates the extraction of specific classes, such as flavan-3-ols and hydroxycinnamic acids. This profile is typically observed in wines produced with longer maceration times, which promote the extraction of these particular compounds [70].

On the other hand, sample three contains only five compounds but successfully represents all the main classes of phenolic compounds analyzed. This balanced yet limited profile suggests a less intensive winemaking process in terms of extraction but one that still preserves a basic diversity of phenols at lower concentrations. In contrast, samples nine and ten had the fewest compounds identified, which may indicate simpler wines with a less complex phenolic composition. This profile could be linked to winemaking conditions with shorter skin contact or less extended aging, resulting in reduced phenolic diversity [20].

### 2.5. Antioxidant Capacity

The antioxidant capacity was evaluated using three assays (FRAP, ABTS, and DPPH). Significant differences between the samples were observed upon analyzing the data presented in Table 4.

The antioxidant capacity assays (FRAP, ABTS, and DPPH) consistently showed that sample five exhibited the highest antioxidant activity, with values of 28.02 ± 1.32, 25.08 ± 0.51, and 25.60 ± 1.82 mmol TE/L, respectively. In contrast, sample two had the lowest antioxidant capacity, with values of 16.58 ± 0.48, 16.04 ± 0.32, and 15.48 ± 0.22 mmol TE/L. These results align with the higher levels of *ortho*-diphenols (ODC) and flavonoids (FC) found in samples five and six, suggesting that the presence of these phenolic compounds plays a significant role in the observed antioxidant capacity.

The higher antioxidant capacities in samples five and six can be attributed to their elevated concentrations of polyphenols, including ODC and FC, both of which are known for their strong antioxidant properties. Previous studies have shown that wines with higher polyphenol content typically exhibit greater antioxidant potential. The presence of other phenolic compounds, such as tannins and flavonols, further enhances the antioxidant capacity of these wines. Tannins, for example, contribute to antioxidant activity by scavenging free radicals and chelating metal ions, while flavonols also play a role in neutralizing reactive oxygen species [69,72].

The high polyphenol content in samples five and six can be directly linked to the winemaking conditions, specifically the extended aging in wood. This aging process promotes the extraction and preservation of phenolic compounds due to the interaction between the wine and the compounds released from the barrel’s wood. Research has shown that prolonged wood aging leads to higher concentrations of phenolic compounds, which are crucial for enhancing the antioxidant capacity of the wine [68].

In contrast, sample two exhibited a lower antioxidant capacity, which can be attributed to a shorter period of wood aging. The limited contact with wood restricted the extraction and stabilization of phenolic compounds, resulting in a lower concentration of antioxidants. This difference in aging time likely led to the reduced presence of polyphenols, such as ODC and FC, in sample two. Additionally, the winemaking techniques employed for sample two, such as fermentation time and temperature, may have also influenced the phenolic profile, further contributing to its lower antioxidant capacity [73].

Furthermore, a comparison with the study by Cristino et al. (2013) [74], which reported higher DPPH and ABTS values (21.70 ± 1.30 and 18.88 ± 0.13 mmol TE/L, respectively), suggests that variations in winemaking techniques, such as differences in maceration, fermentation conditions, and wood aging, could explain the discrepancies observed between the two studies.

In summary, the antioxidant capacity of wine is strongly influenced by the concentration and composition of phenolic compounds, such as ODC, FC, tannins, and flavonols. The higher antioxidant activity in samples five and six can be attributed to their extended aging in wood, which promoted the extraction of these compounds. In contrast, the low antioxidant capacity observed in sample two is primarily due to the shorter aging period, which limited the extraction and stabilization of phenolic compounds.

### 2.6. Pearson Correlation

**Figure 4:**
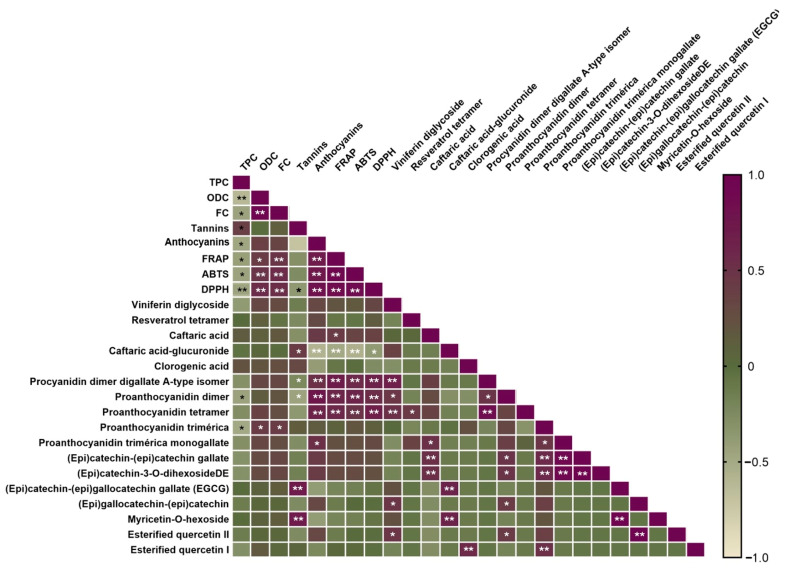
Pearson correlation between phenolic content (TP, ODC, FC), antioxidant capacity, and phenolic compounds present in wine samples by RP–HPLC–DAD–ESI-MS/MS. Statistically significant correlations: * *p* < 0.05, ** *p* < 0.01.

In order to establish the correlation between all parameters analyzed, a Pearson correlation was performed, as shown in Figure 4.

This correlation matrix provides a robust analysis of the relationships between phenolic compounds, antioxidant capacity, and phenolic content in wine. Some key takeaways from the analysis: (1) total phenolic content (TPC): The positive correlations between TPC and the antioxidant assays (FRAP, ABTS, DPPH) strongly suggest that phenolic compounds are directly tied to the antioxidant potential of the wine. The correlation with proanthocyanidins further supports their role in both the phenolic profile and the antioxidant properties of the wine. (2) Antioxidant assays: The fact that the FRAP, ABTS, and DPPH methods correlate well with each other indicates that they are reliable in measuring antioxidant capacity. The consistency between these methods provides confidence that the measured antioxidant activities are representative of the wine’s true antioxidant potential. (3) Caftaric acid: Its strong correlation with both FRAP and TPC underlines its importance in antioxidant activity. This acid is a key phenolic compound in wine and contributes significantly to the wine’s overall antioxidant properties. (4) Procyanidins and proanthocyanidins: These tannin compounds play a critical role in the wine’s antioxidant activity. Their positive correlations with TPC and antioxidant assays emphasize their importance in the wine’s structure, astringency, and health benefits. (5) Flavonoids: Compounds like (epi)catechin gallate and (epi)gallocatechin gallate also show positive correlations with antioxidant activity, reinforcing their significant contribution to the antioxidant properties of the wine. (6) Tannins and anthocyanins: While tannins show a strong relationship with antioxidant capacity, anthocyanins, despite contributing to color, seem to have a less prominent role in antioxidant activity in this analysis. This could be due to the particular focus of this study on antioxidant properties, where other phenolic compounds may play a more direct role.

The correlation matrix clearly highlights the strong influence of certain phenolic compounds—especially proanthocyanidins, caftaric acid derivatives, and catechins—on the antioxidant capacity of Touriga Nacional wines. This analysis not only reinforces the health benefits of phenolic-rich wines but also provides a deeper understanding of the specific compounds responsible for these benefits. By focusing on the phenolic composition, the study contributes valuable insights into the functional qualities of wine, which can be leveraged for both health benefits and winemaking practices.

## 3. Materials and Methods

### 3.1. Chemicals

Potassium hydroxide (KOH), Folin–Ciocalteu’s reagent, gallic acid (3,4,5-trihydroxybenzoic acid), acetic acid (CH_3_COOH), formic acid (HCOOH), and sodium hydroxide (NaOH) were obtained from Panreac Química SLU (Barcelona, Spain). Hydrochloric acid (HCl), sodium nitrite (NaNO_2_), aluminum chloride (AlCl_3_), and sodium carbonate (Na_2_CO_3_) were from Merck (Darmstadt, Germany). Methanol (CH_3_OH) and ABTS^•+^ (2,2-azino-bis(3-ethylbenzothiazoline-6-sulfonic acid) diammonium salt were purchased from VWR (Carnaxide, Lisbon, Portugal). Sodium molybdate (Na_2_MoO_4_) was acquired from Chem-Lab N.V. (Zedelgem, Belgium). Moreover, catechin (C_15_H_14_O_6_), Trolox (6-hydroxy-2,5,7,8-tetramethylchroman-2-carboxylic acid), potassium persulfate (K_2_S_2_O_8_), TPTZ (2,4,6-Tripyridyl-s-Triazine), and iron (III) chloride (FeCl_3_) were sourced from Sigma-Aldrich (Steinheim, Germany). In the case of DPPH^•^ (2,2-diphenyl-1-picrylhydrazyl radical), it was obtained from Alfa Aesar (Porto Salvo, Portugal). While acetonitrile was supplied by J.T. Baker (Philipsburg, NJ, USA). Distilled water from Millipore (Bedford, MA, USA) was used for all extractions and analyses.

### 3.2. Sampling

The sample consisted of 10 single-varietal 2019 Touriga Nacional wines from the Cima Corgo sub-region of the Douro region, with the winemaking process for each wine detailed in Table 5. The only variable under study was the external factors that could explain the variability in the phenolic composition.

#### Sampling Preparation

All samples were diluted at a 1:100 ratio, with 1 mL of the sample diluted to 100 mL of distilled water for the determination of phenolic content and antioxidant capacity. Each sample was prepared in triplicate. For tannin determination, the samples were diluted at a 1:5 ratio, also in triplicate. Anthocyanin determinations were carried out using undiluted samples.

### 3.3. Determination of Phenolic Content

The phenolic content of the wine samples was analyzed for total phenolics, *ortho*-diphenols, and flavonoids through spectrophotometric techniques, following the protocols outlined by Leal, C., et al. [75], with minor modifications. Absorbance was measured using a spectrophotometer (Thermo Spectronic Genesys 10-S, Rochester, New York, NY, USA) with cuvettes (10 × 35 mm). The data were expressed as the mean of three replicates (*n* = 3) ± standard deviation.

#### 3.3.1. Total Phenolic Content

The total phenolic content was determined using the Folin–Ciocalteu method as previously described by our research group [76]. For that, 1 mL of the sample/standard was mixed with 0.5 mL of Folin–Ciocalteu reagent, adding 2 mL of Na_2_CO_3_ (7.5%, *w*/*v*) and 6.5 mL of H_2_O. The mixture was incubated at 70 °C for 30 min, protected from light. Absorbance readings were taken at 750 nm, using gallic acid as a standard (concentration range: 5–200 mg/L).

The results were reported in milligrams of gallic acid equivalents per liter of wine (mg GAE/L).

#### 3.3.2. *Ortho*-Diphenols Content

The *ortho*-diphenols content was determined by mixing 160 µL of the sample/standard solution with 40 µL of Na_2_MoO_4_ (5%, *w*/*v*). The mixtures were vortexed and then incubated at room temperature, protected from light, for 15 min. Absorbance was measured at 375 nm using a gallic acid standard curve (concentration range: 5–200 mg/L).

The results were expressed as milligrams of gallic acid equivalents per liter of wine (mg GAE/L).

#### 3.3.3. Flavonoid Content

The flavonoid content was determined by mixing 24 µL of the sample/standard with 28 µL of NaNO_2_ (5.0%, *w*/*v*). After 5 min, 28 µL of AlCl_3_ (10.0%, *w*/*v*) were added, and the mixture was allowed to react for 6 min. Subsequently, 120 µL of NaOH (1.0 M) was added, followed by shaking for 30 s. The absorbance of the resulting solution was measured at 510 nm. The concentration of flavonoids was calculated using a catechin standard curve (concentration range: 5–200 mg/L).

The results were expressed as milligrams of catechin equivalents per liter of wine (mg CE/L).

### 3.4. Determination of Total Anthocyanins

The total anthocyanin content of the wine samples was determined using the differential pH method. In two centrifuge tubes, 1 mL of the sample and 1 mL of 0.1% HCl ethanolic solution were added. To one centrifuge tube, 10 mL of buffer solution (pH 3.5) was added, and to the other centrifuge tube, 10 mL of buffer solution (pH 0.6) was added. Each assay was performed in duplicate.

Finally, the absorbance was measured at 520 nm.

All results are expressed in milligrams of malvidine per liter of sample (mg MALV/L) by applying the methodology described by Lee J., et al. [77].

### 3.5. Determination of Total Tannins

The total tannin content was determined using methylcellulose precipitable (MCP) tannin assay.

The MCP tannin assay was performed based on the method described by Mercurio, Meagan D., et al. [78], with slight modifications. A 0.04% *w*/*v* methylcellulose solution was prepared according to the manufacturer’s instructions (Sigma-Aldrich, Castle Hill, NSW, Australia).

In one centrifuge tube designated as the control tube, 1 mL of each sample was mixed with 2 mL of saturated ammonium sulfate solution and 6.5 mL of distilled water. After stirring, the mixture was left to rest for 10 min at room temperature. Finally, it was centrifuged for 5 min at 4000 rpm using a Sigma centrifuge (Steinheim, Germany), and the absorbance was read at 280 nm.

In another centrifuge tube, 1 mL of each sample was mixed with 3 mL of methylcellulose (0.04%). After stirring, the mixture was left to rest for 2 to 3 min. After that, 2 mL of saturated ammonium sulfate solution and 4 mL of distilled water were added. The mixture was stirred again and left to rest for 10 min at room temperature. Finally, it was centrifuged for 5 min at 4000 rpm, and the absorbance was read at 280 nm.

All results were expressed in milligrams of epicatechin per liter of wine (mg EPICAT/L).

### 3.6. (Poly) Phenolic Profile

The quantitative (poly) phenolic profile of the 10 wines was achieved by applying the methodology described by Costa-Pérez, A., et al. [79], with some modifications.

Chromatographic separations were carried out using a Thermoscientific VANQUISH C18 column (150 2.1 mm, 2.2 µM particle size; Thermo Fisher Scientific Inc., Vilnius, Lithuania). The chromatographic resolution of the phenolic profile was achieved using deionized water/formic acid (99.9:0.1, *v*/*v*) (A) and acetonitrile/formic acid (99.9:0.1, *v*/*v*) (B) as chromatographic solvents using the following gradient (Time, %B): (0, 10%), (20, 60%), (20.1, 10%), and (25, 10%). The flow rate was 0.3 mL/min, and the injection volumes were 7 µL. The HPLC system was equipped with a Vanquish-LTQ-XL-ThermoScientific diode array and a mass detector in series (Thermo Scientific Dionex UltiMate 3000 Series, Bremen, Germany). It consisted of a quaternary SD, RS, BM, and BX pump, a WPS-3000 autosampler, a G1322A degasser, and an Electrochemical photodiode array detector controlled by Xcalibur software version 08.03 (Agilent Technologies, Waldbronn, Germany). Spectroscopic data from all peaks were accumulated in the range of 240–600 nm, and the spectral data were recorded at 280, 330, and 370 nm. The mass detector was a G2445A Ion-Trap Mass Spectrometer equipped with an electrospray ionization (ESI) system and controlled by LTQ Tune software version 4.1 (Agilent, Waldbronn, Germany). Nitrogen was used as a nebulizing gas at a pressure of 60 psi, and the flow was adjusted to 11 L/min. The heated capillary and voltage for ionization were maintained at 350 C and 5 kV, respectively. Collision-induced fragmentation experiments were performed in the ion trap using helium as a collision gas, with voltage ramping cycles from 0.3 up to 2 V. The full scan mass covered the range from *m*/*z* 100 up to *m*/*z* 2000. Mass spectrometry data were acquired in the negative ionization mode. Total ion chromatograms were recorded as full-scan mass spectra (MS). The identification of the individual phenolic compounds was performed by analyzing the retention time (min), parent ions, and fragmentation patterns in comparison with authentic standards and, when they were not available, descriptions available in the literature.

### 3.7. Determination of Antioxidant Capacity

The radical scavenging capacity of the extracts was assessed using two methods: the DPPH (2,2-diphenyl-1-picrylhydrazyl) and the ABTS (2,2′-azino-bis (3-ethylbenzothiazoline-6-sulfonic acid)) assays. Additionally, the reducing activity was measured using the ferric-reducing antioxidant power (FRAP) assay. These methodologies were adapted for microscale using 96-well microplates (PrimeSurface MS-9096MZ, Frilabo, Maia, Portugal), according to Costa, R.D., et al. [76]. Absorbance measurements were conducted using a microplate reader (Multiskan GO Microplate Photometer, Thermo Fisher Scientific, Vantaa, Finland). The results were expressed as the mean ± standard deviation.

#### 3.7.1. DPPH Radical Scavenging Assay

Wines or standard solutions (10 µL) were mixed with 190 µL DPPH working solution and incubated for 30 min in darkness at room temperature. The absorbance was measured at 520 nm. Methanol/H_2_O (70:30, *v*/*v*) was used as a blank.

The scavenging capacity of the samples was determined using Trolox as a reference (0.039 to 0.313 mmol/L). The results were expressed as millimoles of Trolox equivalents per liter (mmol TE/L). The percentage of inhibition and Trolox Equivalent Antioxidant Capacity (TEAC) of samples was calculated as follows:

% inhibition = 100 × (Abs520 blank − Abs520 sample)/Abs520 blank(1)

(2) $$\mathrm{TEAC}\;(\mathrm{mmol}\;\mathrm{TE}/L)=\frac{\%inhibition-b}{a}$$

where *a* is the slope of the standard curve (y = ax + b); *b* is the y-intercept.

#### 3.7.2. ABTS Radical Scavenging Assay

The ABTS^●+^ scavenging capacity assessment of the wine samples was conducted by mixing the diluted samples (12 µL) with 188 µL of the ABTS working solution (prepared by combining 5 mL of ABTS stock solution (7.0 mM in H_2_O) with 88 µL of potassium persulfate (148 mM) and diluted with sodium acetate buffer (20 mM, pH 4.5). Absorbance was measured at 734 nm after a 30 min reaction. The radical scavenging capacity was quantified as mmol TE/g dw using a Trolox standard curve (0.034 to 0.285 mmol/L).

Percent inhibition and TEAC were calculated based on absorbance readings, comparing sample absorbance to controls and blanks, following the equations:

% inhibition = 100 × (Abs734 blank − Abs734 sample)/Abs734 blank(3)

(4) $$\mathrm{TEAC}\;(\mathrm{mmol}\;\mathrm{TE}/L)=\frac{\%inhibition-b}{a}$$

where *a* is the slope of the standard curve (y = ax + b); *b* is the y-intercept.

#### 3.7.3. FRAP Assay

The FRAP assay used a daily-prepared FRAP working solution: 1 volume of TPTZ (10 mM in HCl), 1 volume of FeCl_3_ (20 mM in water), and 10 volumes of acetate buffer (pH 3.6). In each assay, 20 µL of wine samples were mixed with 180 µL of the pre-warmed FRAP solution at 37 °C for 10 min. Subsequently, the mixtures were incubated in darkness at 37 °C for 30 min. The absorbance was then measured at 593 nm using a microplate reader. Trolox standards (0.020 to 1.250 mmol/L) were employed to construct a calibration curve.

Results were expressed in millimoles of Trolox equivalent per liter (mmol TE/L).

### 3.8. Statictical Assay

Regression equations (y = ax ± b), coefficient of determination (R^2^), adjusted R^2^, and correlation coefficient (r) were also calculated for each method. The data obtained were checked for normality, followed by variance analysis (ANOVA) and a multiple range test (Tukey’s test/*t* student test) for a *p*-value < 0.05, using

JMP Statistics 17.1.0 software (JMP, Cary, NC, USA). To understand the nature and degree of the interrelationships between phenolic composition and their antioxidant capacity, a correlation analysis (Pearson’s coefficient, r-value) was carried out, commonly used to express the strength of the relationship between two continuous variables using the software GraphPad Prism (version 9.0.1, GraphPad Software, San Diego, CA, USA).

**Figure 5:**
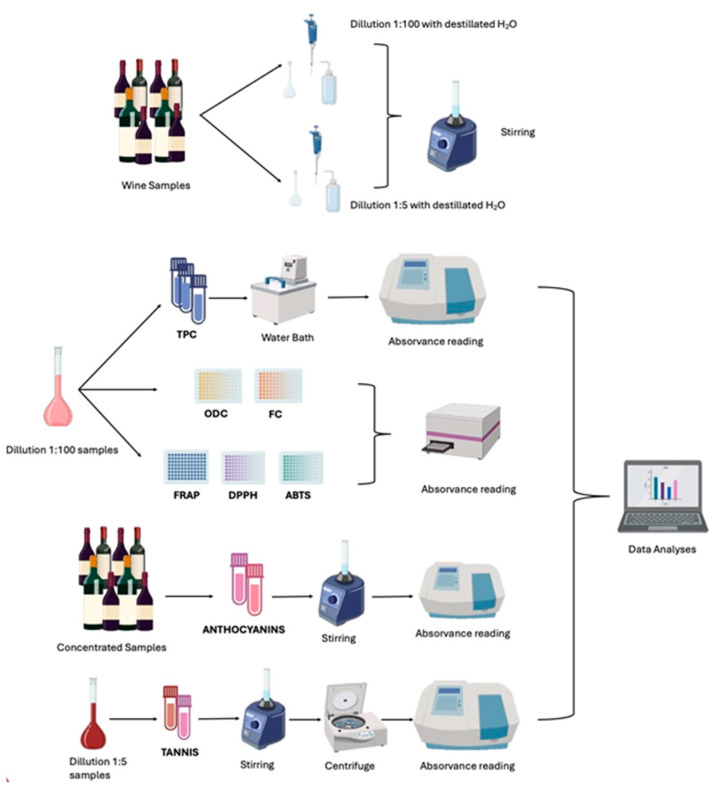
Schematic representation of the methodologies used.

All the methodologies are represented in Figure 5.

## 4. Conclusions

This study demonstrates that the winemaking process plays a crucial role in shaping the phenolic composition and antioxidant properties of Touriga Nacional wines. Samples aged for longer periods in wooden barrels exhibited higher concentrations of phenolic compounds, particularly flavonoids and *ortho*-diphenols, which are strongly correlated with antioxidant capacity. Wines subjected to extended maceration times also showed enhanced phenolic complexity, further emphasizing the importance of vinification techniques in defining the quality of wines. The use of RP-HPLC-DAD-ESI-MS/MS provided a detailed characterization of the phenolic compounds, revealing distinct phenolic profiles across the samples. These findings highlight the potential of phenolic profiling as a valuable tool for wine authentication and quality control, enabling differentiation based on production methods. Overall, this research underscores the significance of controlled winemaking techniques in optimizing phenolic content and antioxidant properties, thereby enhancing the overall quality and market value of Touriga Nacional wines. Future studies could investigate the impact of different oak barrel types and storage conditions on the stability of phenolic compounds and their influence on sensory attributes.

## Figures and Tables

**Table 1:** Phenolic content: TPC, ODC, and FC.

Samples	TPC (mg GAE/L)	ODC (mg GAE/L)	FC (mg CE/L)
1	1009.01 ± 87.92 ^e^	3023.06± 81.29 ^b^	1607.25 ± 100.43 ^b,c^
2	1740.24 ± 131.82 ^c^	2263.20 ± 27.38 ^d^	1281.88 ± 93.82 ^d^
3	1818.32 ± 82.12 ^c^	2811.59 ± 23.87 ^b,c^	1715.94 ± 63.54 ^b^
4	1734.23 ± 193.05 ^c^	2837.87 ± 84.39 ^b,c^	1456.04 ± 71.49 ^c,d^
5	1029.28 ± 3.19 ^e^	3677.78 ± 166.7 ^a^	2115.22 ± 107.60 ^a^
6	1043.54 ± 78.68 ^e^	3505.74 ± 168.23 ^a^	2154.11 ± 116.58 ^a^
7	1464.72 ± 74.78 ^d^	2595.34 ± 65.7 ^c^	1692.75 ± 112.73 ^b,c^
8	2983.48 ± 227.93 ^a^	2816.37 ± 101.02 ^b,c^	2054.11 ± 156.32 ^a^
9	3308.56 ± 245.26 ^a^	518.28 ± 38.02 ^e^	730.44 ± 7.69 ^e^
10	2076.58 ± 66.36 ^b^	431.66 ± 27.38 ^e^	707.73 ± 41.64 ^e^

CE: Catechin equivalents, GAE: Gallic acid equivalents, (*n* = 3 per sample). Data are present as mean ± SD. Different letters in the same column correspond to significant differences between samples (*p* < 0.05). ANOVA followed by a post hoc Tukey test.

**Table 2:** Quantification of total anthocyanins and tannins.

Samples	Anthocyanins (Malv/L)	Tannins (EPICAT/L)
1	175.60 ± 6.79 ^f^	97.28 ± 4.91 ^c^
2	91.40 ± 8.77 ^h^	79.43 ± 1.28 ^d,e^
3	94.40 ± 9.05 ^h^	157.79 ± 6.62 ^a^
4	440.00 ± 3.40 ^c^	67.05 ± 2.52 ^e,f^
5	556.00 ± 1.70 ^a^	67.90 ± 4.81 ^e,f^
6	497.20 ± 21.50 ^b^	77.23 ± 5.46 ^d,e,f^
7	441.20 ± 3.96 ^c^	65.84 ± 3.29 ^f^
8	138.00 ± 1.70 ^g^	124.92 ± 9.50 ^d^
9	228.60 ± 4.81 ^e^	93.85 ± 0.35 ^c^
10	246.20 ± 1.98 ^d^	85.23 ± 3.09 ^c,d^

EPICAT: Epicatechin, Malv: Malvidine, (*n* = 2 per sample for anthocyanins and *n* = 3 per sample for tannins). Data are present as mean ± SD. Different letters in the same column correspond to significant differences between samples (*p* < 0.05). ANOVA followed by a post hoc Tukey test.

**Table 3:** Identification and quantification of phenolic compounds present in wine samples by RP-HPLC-DAD-ESI-MS/MS.

Rt	λ (nm)	[M–H]−, *m*/*z*	Identified Compounds	Quantification (mg/ L)									
				1	2	3	4	5	6	7	8	9	10
**Stilbens**													
1.34	280	777	Viniferin diglycoside	ND	38.609 ± 0.240 ^c^	36.388 ± 0.646 ^c^	ND	75.836 ± 1.564 ^a^	ND	56.939 ± 0.760 ^b^	ND	ND	ND
15.13	320	905	Resveratrol tetramer	ND	ND	ND	1.197 ± 0.104 ^a^	ND	ND	ND	ND	ND	ND
			**Total**	ND	38.609 ± 0.240	36.388 ± 0.646	1.197 ± 0.104	75.836 ± 1.564	ND	56.939 ± 0.760	ND	ND	ND
**Hydroxycinnamic acids**													
3.49	280	311	Caftaric acid	ND	1.901 ± 0.076 ^b,c^	ND	1.680 ± 0.021 ^c^	3.938 ± 0.117 ^b^	4.734 ± 0.096 ^a^	ND	1.909 ± 0.057 ^b^	4.631 ± 0.032 ^a^	ND
7.33	320	487	Caftaric acid-glucuronide	ND	0.934 ± 0.011 ^a^	0.831 ± 0.341 ^a^	ND	ND	ND	ND	ND	ND	ND
11.01	280	353	Clorogenic acid	0.683 ± 0.312 ^a^	ND	ND	ND	ND	ND	ND	0.868 ± 0.019 ^a^	ND	ND
			**Total**	0.683 ± 0.312	2.835 ± 0.435	0.831 ± 0.341	1.680 ± 0.021	3.938 ± 0.117	4.734 ± 0.096	ND	2.777 ± 0.038	4.631 ± 0.032	ND
**Flavan-3-ols**													
2.37	280	879	Procyanidin dimer digallate A-type isomer	ND	ND	ND	ND	1.838 ± 0.442	ND	ND	ND	ND	ND
5.06	280	577	Proanthocyanidin dimer	ND	ND	ND	ND	3.370 ± 0.152 ^a^	3.391 ± 0.098 ^a^	3.057 ± 0.062 ^b^	ND	ND	2.694 ± 0.109 ^c^
5.81	280	1153	Proanthocyanidin tetramer	ND	ND	ND	4.956 ± 0.146 ^b^	8.762 ± 0.076 ^a^	ND	ND	ND	ND	ND
5.83	280	865	Proanthocyanidin trimer	8.548 ± 0.069 ^b^	ND	4.561 ± 0.136 ^d^	ND	ND	9.362 ± 0.151 ^a^	6.833 ± 0.228 ^c^	1.003 ± 0.044 ^e^	ND	ND
7.20	280	1017	Proanthocyanidin trimer monogallate	ND	ND	ND	3.404 ± 0.157 ^b^	ND	7.765 ± 0.073 ^a^	ND	ND	ND	ND
8.83	520	729	(Epi)catechin-(epi)catechin gallate	ND	ND	ND	ND	ND	2.387 ± 0.010 ^a^	ND	ND	ND	ND
10.40	280	613	(Epi)catechin-3-*O*-dihexoside	ND	ND	ND	ND	ND	7.240 ± 0.008 ^a^	ND	ND	ND	ND
10.40	280	745	(Epi)catechin-(epi)gallocatechin gallate (EGCG)	ND	ND	5.879 ± 0.016 ^a^	ND	ND	ND	ND	ND	ND	ND
11.62	520	593	(Epi)gallocatechin-(epi)catechin	ND	ND	ND	ND	ND	ND	0.484 ± 0.020 ^a^	ND	ND	ND
			**Total**	8.548 ± 0.069	ND	10.44 ± 0.076	8.36 ± 0.151	13.97 ± 0.223	30.145 ±0.068	10.374 ± 0.31	1.003 ± 0.044	ND	2.694 ± 0.109
**Flavonols**													
9.21	280	479	Myricetin-*O*-hexoside	ND	ND	3.390 ± 0.034 ^a^	ND	ND	ND	ND	ND	ND	ND
10.90	320	1131	Esterified quercetin II	ND	ND	ND	ND	ND	ND	ND	ND	ND	ND
14.75	360	301	Esterified quercetin I	2.028 ± 0.015 ^a^	ND	ND	ND	ND	ND	0.48 ± 0.00 ^b^	ND	ND	ND
			**Total**	2.028 ± 0.015	ND	3.390 ± 0.034	ND	ND	ND	0.48 ± 0.00	ND	ND	ND

ND: Not detected, Rt: Retention time. In the same row, different letters correspond to significant differences between samples (*p* < 0.05), according to ANOVA followed by a post hoc Tukey test. Data are present as mean ± SD (*n* = 2 per sample).

**Table 4:** Determination of antioxidant capacity.

Samples	FRAP (mmolTE/L)	ABTS (mmolTE/L)	DPPH (mmolTE/L)
1	21.46 ± 0.30 ^b,c^	20.74 ± 0.62 ^b^	19.22 ± 0.78 ^c,d^
2	16.58 ± 0.48 ^d^	16.04 ± 0.32 ^c^	15.48 ± 0.22 ^e^
3	19.17 ± 1.06 ^c,d^	19.40 ± 0.82 ^b^	19.10 ± 1.27 ^c,d^
4	20.49 ± 0.62 ^c^	20.33 ± 0.96 ^b^	21.22 ± 0.44 ^b,c^
5	28.02 ± 1.32 ^a^	25.08 ± 0.51 ^a^	25.60 ± 1.82 ^a^
6	24.48 ± 1.99 ^a,b^	23.67 ± 1.79 ^a^	22.79 ± 1.72 ^a,b^
7	19.96 ± 1.60 ^c,d^	20.53 ± 1.19 ^b^	19.88 ± 1.64 ^b,c,d^
8	20.54 ± 1.70 ^c^	20.13 ± 1.44 ^b^	16.57 ± 0.88 ^d,e^
9	19.96 ± 1.60 ^c,d^	19.23 ± 0.06 ^b^	17.71 ± 1.28 ^d,e^
10	21.33 ± 0.42 ^b,c^	20.04 ± 0.37 ^b^	17.92 ± 0.77 ^c,d,e^

TE: Trolox equivalents (*n* = 3 per sample). Data are present as mean ± SD. Different letters in the same column correspond to significant differences between samples (*p* < 0.05). ANOVA followed by a post hoc Tukey test.

**Table 5:** Technical Characteristics of the Samples.

Samples	Technical Characteristics	Potential Influencing Factors
1	Alcoholic fermentation in a “lagar”, with **traditional treading**	Traditional treading Aging for 12 months in wood barrel
	The wine was placed in **casks**, where the **malolactic fermentation** occurred, and aging was performed in the same **wood barrel for 12 months**	
2	Alcoholic fermentation in **stainless tanks**	Aging for 18 months in wood barrel
	**Aging for 18 months in wood barrel**	
3	**Cold pre-fermentation**	Maceration Aging in wood barrel new and neutral
	Spontaneous alcoholic fermentation, with **maceration for 32 days**	
	**18 months in wood barrel** (75% **new**, 25% **neutral**)	
4	Alcoholic fermentation **stainless tanks**	Soft Pressing Aging for 24 months in wood barrel
	After malolactic fermentation, **soft pressing** was performed	
	**24 months in wood barrel**	
5	Partially destemmed grapes	Soft pressing Aging for 36 months in wood barrel
	Alcoholic fermentation in **stainless tanks**	
	After malolactic fermentation, **soft pressing** was performed	
	**36 months in wood barrel**	
6	Alcoholic fermentation in a “lagar”, with **traditional treading**	Aging for 36 months in wood barrel Aging for 24 months in bottle
	After malolactic fermentation, soft pressing was performed	
	**Malolactic fermentation in wood barrel**	
	**36 months in wood barrel** and **24 months in bottle**	
7	Alcoholic fermentation and malolactic fermentation simultaneously in **stainless tanks** for 12 days	Post-fermentation maceration Soft pressing Aging for 18 months in wood barrel Aging for 36 months in stainless tanks
	**Post-fermentation maceration for 30 days**	
	**Soft pressing**	
	**18 months in wood barrel** and **36 months stainless tanks**	
8	**Maceration in “microlagares”** (T: 26–28 °C)	Maceration Aging for 9 months in wood barrel
	**9 months in wood barrel**	
9	Alcoholic fermentation in **stainless tanks**, for **8 days**	Aging for 12 months in wood barrel
	Malolactic fermentation in **stainless tanks**, for **3 weeks**	
	**12 months in wood barrel**	
10	**Grapes selection**	Soft pressing Aging for 18 months in wood barrel
	Alcoholic fermentation **stainless tanks**	
	After malolactic fermentation, **soft pressing** was performed	
	**18 months in wood barrel**	

## Data Availability

The original contributions presented in this study are included in the article. Further inquiries can be directed to the corresponding author.
